# Experiences of Nursing Interns in the Neonatal Intensive Care Unit in Saudi Arabia: A Phenomenological Study

**DOI:** 10.7759/cureus.74893

**Published:** 2024-12-01

**Authors:** Ahmad Ismail, Rawan Gashgari

**Affiliations:** 1 Nursing Department, Fakeeh College for Medical Sciences, Jeddah, SAU; 2 Neonatal Intensive Care Nursing Program, Nursing Department, Fakeeh College for Medical Sciences, Jeddah, SAU

**Keywords:** clinical placement, lived experience, nicu, nursing interns, saudi arabia, training

## Abstract

Background: Clinical training in the neonatal intensive care unit (NICU) is a unique experience due to the specialty and complex care provided for critically ill neonates. There is a lack of research exploring nursing interns' experience in the NICU.

Purpose: This study aimed to explore the nursing interns’ experience regarding their clinical placement in the NICU in Saudi Arabia.

Method: A phenomenological study was used to understand the lived experience of nursing interns in the NICU. Data were collected through two semi-structured focus group interviews. The first focus group included eight nursing interns, and the second group included seven. These interns completed their NICU placement. Thematic analysis was employed to identify key themes from the data.

Results: Four main themes emerged from the analysis: (1) pleasant experiences in the NICU training, (2) sources of encouragement in the NICU training, (3) barriers to effective learning in the NICU, and (4) assessment and growth.

Conclusion: Supportive NICU staff and practical involvement enhanced effective learning, while administrative tasks and improper use of interns’ time posed significant barriers. Revising the nursing interns’ training program in the NICU is needed to reduce non-educational tasks and improve assessment methods to better reflect interns' clinical performance.

## Introduction

Bachelor of Nursing programs in Saudi Arabia are designed as four years of academic study followed by one year of internship training [1]. The internship year is the year that follows the four-year theoretical and clinical study in nursing school and precedes registration in the governmental body for nursing (Saudi Commission for Health Specialties). Completing the internship year is essential to being a registered nurse in Saudi Arabia [2]. Nursing interns complete more independent clinical training this year by rotating in different hospital areas [3,4]. Clinical nursing practice during the internship is crucial, as it links what the nursing students have studied theoretically and applies nursing skills with more autonomy [4-9]. The internship year comprises 52 weeks of training, including four weeks of holidays [4,10,11]. Nursing interns undertake their rotations and clinical placements in several nursing departments, including the neonatal intensive care unit (NICU).

Training in the NICU is both rewarding and distressing [12-15]. Nursing students may feel distressed and apprehensive due to the complexity of care provided to neonates and the advanced equipment used in the NICU [12-14,16]. To minimize any complications or errors, healthcare professionals in the NICU may allow nursing interns to practice only minimal hands-on skills [4,7,8,14], although clinical practice is vital for acquiring nursing competencies [14]. Understanding the experiences of nursing interns in the NICU is crucial for optimizing the benefits of their training and fostering a more positive perception of their NICU experience.

Several studies have been conducted in Saudi Arabia regarding internship years [6,8,9,17,18]. However, there is a lack of research exploring nursing interns’ perceptions of their experiences in the NICU clinical placement. This study explored the nursing interns’ experience to understand their perception of their clinical placement in the NICU.

## Materials and methods

Study design

This study utilized a qualitative, descriptive phenomenological research design to deeply understand the nursing interns’ experience regarding their training in the NICU.

Setting

Data were collected from nursing interns who completed their internships at Dr. Soliman Fakeeh Hospital in Jeddah, Saudi Arabia. The interview sessions were conducted in the hospital meeting rooms and at Fakeeh College Medical Sciences. These rooms were quiet and free of distractions.

Sampling technique and sample size

This study recruited a convenience sample of 15 nursing interns out of 50, divided into two focus groups. The first group included eight interns, and the second group had seven. The interns’ coordinator was contacted to facilitate access to the nursing interns who had a clinical placement in the NICU for at least one week in the past year. Seven to eight participants were chosen for each interview and invited to attend the focus-group session. Focus group interviews were conducted and guided by the interviewer using a topic guide. A focus group is an extended way of the interview method, a more specific in-depth group interview with discussion. The topic was explored in a semi-structured and organized manner with the help of a facilitator or moderator. Inclusion criteria were nursing interns who had at least one month of rotation in the NICU and who could communicate in English. 

Data collection procedure

The data were collected from 10 January 2024 to 30 May 2024 through two in-depth focus group interviews using a semi-structured interview guide. Focus group interviews help in understanding group dynamics, generating ideas, exploring social norms, and gathering a diverse range of perspectives in a relatively short amount of time. The group interaction and discussion often result in richer and more nuanced data, especially when studying collective attitudes or behaviors. The interviews were digitally recorded and transcribed verbatim. All participants were informed about the date, time, and venue of the focus group sessions in advance. A moderator facilitated the focus group interactions using a set of previously planned, related open-ended questions. Each session lasted one hour and followed a semi-structured pattern, using the following prepared questions: (1) Tell me about the most pleasant event during your internship in the NICU. (2) What do you think about your internship training in the NICU? (3) What support was provided during the rotation? (4) How did your preceptors/instructors facilitate the learning process? (5) How helpful were the NICU staff to you? (6) What was the role and involvement of your academic faculty during the NICU rotation? (7) What encouraged your learning and helped in your learning experience? (8) What is your favorite/best learning strategy? Explain. (9) Tell me about the barriers that hindered your successful internship in the NICU. (10) What are your final thoughts and comments regarding your internship experience?

Each focus group interview was conducted in four phases: opening, warm-up, main body, and closure. The opening phase served as an icebreaker, introducing the participants and the topic and explaining the purpose and ground rules. The warm-up phase involved asking the least threatening and simplest questions to stimulate group interaction, such as “Tell me about the most pleasant event during your internship in the NICU.”

The main body of the interview consisted of more complex and sensitive questions, eliciting deep and broad responses, such as “Tell me about the barriers that hindered your successful internship in the NICU.” Finally, the facilitator closed the interview with closure-type questions like “What are your final thoughts and comments regarding your internship experience?” This phase also involved summarizing the interview and thanking the participants for their active participation.

Rigor

To ensure the trustworthiness and truthfulness of the findings, this study adhered to the four criteria established by Lincoln and Guba to maintain rigor [19]. The first criterion, credibility, was achieved by accurately and truthfully describing the interns' lived experiences in the NICU placement. This involved prolonged engagement and persistent observation to fully understand the context and minimize potential distortions in the data. Specific actions include (1) spending sufficient time with the interns to understand their situation and build trust and rapport; (2) engaging in peer debriefing through meetings and discussions with an expert qualitative researcher to allow for questions and critique; and (3) performing member checking by regularly verifying data and interpretations with the interns and sharing conclusions with them to ensure accuracy and provide opportunities for additional input.

The second criterion, transferability, was achieved by providing a detailed description of the interns’ experiences. Recruitment continued until data saturation was reached, ensuring completeness and replicability. The primary investigator transcribed the digitally recorded data, which were then reviewed by a professional researcher. Consistency in data collection methods across the two focus groups was achieved using a semi-structured interview with prepared questions to guide the interview and allow for new information. 

Dependability, the third criterion, was maintained through several actions: employing a code-recode procedure where the investigator waited at least two weeks after initial coding to re-evaluate and recode the same data, ensuring consistency in findings. A PhD graduate from Fakeeh College reviewed some transcribed materials to validate the themes, and any new themes identified were acknowledged and considered. Additionally, participants were asked to evaluate the extent to which the findings represented their interpretations and views.

The fourth criterion, confirmability, was ensured by (1) maintaining self-awareness of my role as the primary instrument of the study and (2) documenting additional perceptions and recollections immediately after each interview in a private room. 

Data analysis

Focus group interviews were digitally recorded and transcribed verbatim. Thematic analysis was used to analyze the data [20]. Words, phrases, and statements describing the experiences of nursing interns in the NICU were identified and highlighted. These highlights were used to form themes reflecting their experiences. After thoroughly reading all the transcripts, the data were coded based on relevance. These codes were then refined and grouped into potential themes. A PhD graduate from Fakeeh College reviewed some transcripts, coding, and themes. Any new themes and descriptors suggested were considered. The data and interpretations were then shared with participants to ensure their views were accurately represented. Finally, the themes were defined and named, and a narrative structure with detailed descriptions was created.

Ethics

Ethical approval was obtained from the Ethics Board at Fakeeh College for Medical Sciences (465/IRB/2023) on 23 February 2023. Participants’ rights were ensured. Consent for participation in the research was obtained from all participants, along with their permission for digital recording, which was taken by asking them to sign a form before the interview started. Participants were assured that their names would not be mentioned in the study at all, and each participant was addressed in the study as a number. Data were published using numbers or codes, and the identity of the participants was not disclosed. They were also given the right to withdraw from the study and informed that their data would be kept completely confidential. Participation in this study was voluntary. Participants were assured that their data would be kept confidential and would not be sent to any institutions. Data were kept in a secured cupboard designed for this purpose at Fakeeh College for Medical Sciences. Data will be destroyed after five years of the study completion by “shredding” or “secure deletion” for soft data. No one can access the data except the principal investigator and the college supervisor.

## Results

Data analysis showed various aspects of the interns' training experiences, including pleasant moments, motivating factors, barriers encountered, and evaluation methods used during their NICU training. The results offer valuable insights into the challenges and opportunities faced by nursing interns in this specialized clinical setting, providing implications for education and training programs to enhance the learning experience in the NICU. Four thematic categories structured the nursing interns’ experience in the NICU placement.

Theme 1: Pleasant experiences in the NICU training

Pleasant experiences during NICU training highlight the positive moments the nursing interns encountered during their clinical placement in the NICU. These experiences often contribute significantly to the interns' personal and professional growth, providing them with a sense of accomplishment and fulfillment.

Participants described the most pleasant events during their training, starting with the discharge moments when the patients' families were delighted. When asked, “Tell me about the most pleasant event during your internship in the NICU,” Participant 1 stated, "The discharge moment when I see the parents finally carrying their baby and are so happy and pleased.” Similarly, participant 14 shared, “The baby discharge moment, when the parents finally sign the discharge papers, and the baby is healthy. It is a precious moment.” These moments were especially significant after long-term care of premature babies, as Participant 2 mentioned, “When a preterm who stayed for a long time is finally ready for discharge, I had a sick preterm for a long time, and it was very pleasant when she finally got well.”

Another pleasant moment frequently mentioned is the bonding interns felt with the babies during feeding or routine care. Participant 4 said, “When carrying the babies, I feel like a mother.” Participant 3 said, “All types of feedings, whether it’s NGT or oral feeding, I feel the bonding with the babies in these moments.” Participant 12 also described these moments with warmth and compassion, saying, “There is also a very good feeling when a baby is crying and is calmed immediately when we hold him.”

Additionally, nursing interns found that patients' improving conditions and being weaned from ventilators were also pleasant experiences during their NICU training. Participant 2 stated, “When a baby that was on a ventilator or CPAP is weaned to room air.” The quiet hour was another pleasant experience; Participant 6 described it as “It’s when everyone is quiet, and no one is allowed to speak. I feel relieved and relaxed.” Nursing interns also enjoyed smooth mornings with no meetings or discussions of raised issues, and smooth handovers, as mentioned by Participant 12, “When we hand over to the next shift and the baby is okay, no complications happened” (Table 1).

**Table 1:** Nursing interns’ pleasant experience in the NICU. NICU: neonatal intensive care unit; CPAP: continuous positive airway pressure; NGT: nasogastric tube.

Participant	Phrase	Code
1	"Discharge moment when I see the parents are finally carrying their baby and are so happy and pleased."	Baby discharge joy
14	"The baby discharge moment when the parents finally sign the discharge papers, and the baby is healthy. It is a precious moment"	
2	"When a baby that was on a ventilator or a CPAP is weaned to room air"	Weaning success
3	"When feeding the babies" "All types of feedings whether it’s NGT or oral feeding I feel the bonding with the babies in these moments."	Feeding bonding
4	"When carrying the babies I feel like a mother"	Motherly bonding
12	“There is also a very good feeling, when a baby is crying and is calmed immediately when we hold him”	
5	"When assisting the mother with breastfeeding"	Breastfeeding assistance
6	"The quiet hour" "It’s when everyone is quiet and no one is allowed to speak. I feel relieved and relaxed.”	Quiet hour relief
7	"I like it every time a mother comes and carries her baby and we assist her with everything it’s satisfying"	Assisting mothers
2	"When a preterm who stayed for a long time is finally ready for discharge, I had a sick preterm for a long time and it was very pleasant when she finally got well."	Long-term care and discharge
10	"When we see the patient condition is improving."	Condition improvement
11	"When a morning passes without any problems and morning meetings."	Smooth mornings
14	"Every morning we are gathered for a meeting to discuss an issue that occurred the day or night before. It takes around one hour with ZOOM meeting for those who are not present. So when a morning passes without that it’s a good morning."	Issue-free experience
15	"The normal thing is to keep hearing complaints the abnormal thing is to be happy and not hear any complaints."	
11	"It’s satisfying when we do any nursing practice and the preceptor praises us that it is a good job."	Praise from preceptors
12	"There is also a very good feeling when a baby is crying and is calmed immediately when we hold him."	Calming babies
12	"When we hand-over for the next shift and the baby is ok no complications happened."	Smooth Handover

Theme 2: Sources of encouragement in NICU training

Sources of encouragement in NICU training encompass various factors that foster motivation and positive engagement among nursing interns. These factors included supportive interactions with staff, clear explanations, hands-on practice of nursing skills, involvement in medical rounds, exposure to interesting cases, and facing challenges that stimulate learning.

During interviews, interns emphasized the invaluable support provided by NICU staff. They described a culture where staff members, both preceptors and non-preceptors, were always ready to assist and guide, creating a conducive learning environment most of the time. Participant 8 shared, "The staff used to help us a lot whenever something occurred, and they were very willing to teach us." Furthermore, they noted that even non-preceptor staff members were supportive and willing to answer queries, per Participant 6, when she shared, “They were very helpful, even when I asked other staff who are not preceptors, they were supportive and answered my questions.”

The Clinical Resource Nurse (CRN) in the NICU emerged as a significant source of encouragement. The CRN was described as instrumental in interns' learning journeys through proactive engagement and constructive feedback sessions. "The CRN was amazing," shared Participant 2. Some interns found guidance from CRNs and preceptors particularly beneficial in aligning patient care tasks with their learning objectives. Clear daily objectives and plans provided structure to their training, reducing anxiety and instilling confidence. Participant 14 said, "I liked when the CRN looked at my objectives first, and then she guided me to the patients who would meet my objectives and help me fulfill them. For example, she would say you can finish the blood transfusion in patient one and something else in patient two." Hands-on engagement in nursing procedures was highlighted as a motivating factor, with interns valuing opportunities to participate in tasks like suctioning and intravenous access. "I learn best by doing. When I am involved in procedures, I feel more confident in my skills," shared Participant 13.

Participation in medical rounds, especially in such cases, provided interns with valuable learning opportunities and insights into patient care. They found the rounds engaging and informative, especially when consultants actively involved them in discussions and decision-making processes. Participant 7 shared, “Whenever we attended rounds, we found it very interesting and learned a lot about the cases.” Participant 14 mentioned an interesting story that happened during the rounds, saying, “There was a baby that I thought that there is something abnormal in his mouth and his feeding. During the round, my preceptor was not present, so I was the one answering the questions about the baby. I addressed my concern so the doctor asked for a re-assessment, head-to-toe, and they discovered that the baby had a cleft palate; no one noticed it before.”

Challenges and assignments that required critical thinking and comprehensive understanding were viewed as stimulating learning experiences. Interns expressed a preference for tasks that pushed their intellectual boundaries, emphasizing the importance of being challenged for effective learning and retention. As mentioned by Participant 5, “When I am asked to look for the answers, and I keep looking until I understand it. I learn best when I am challenged.” One more encouraging thing was that they found NICU-specific information easy to grasp. Interns appreciated the manageable complexity of medication knowledge in the NICU. They found comfort in dealing with common medications like caffeine and Survanta, which were perceived as comprehensible and easy to understand, facilitating their learning process. Participant 6 stated, “The number of common medications is not too much and not too difficult to study, caffeine, Survanta, and other medications that are easy to understand.”

Receiving gratitude and appreciation from patient families positively impacted interns' morale and motivation. Participant 4 mentioned, “Babies’ mothers who thanked us and said nice words and prayers, this was very encouraging.” They found solace and encouragement in the heartfelt expressions of gratitude from parents, fostering a sense of fulfillment and purpose in their roles (Table 2).

**Table 2:** Sources of encouragement for nursing interns in the NICU. NICU: neonatal intensive care unit; CRN: Clinical Resource Nurse.

Participant	Phrase	Code
8	"The staff used to help us a lot whenever something occurred"	Staff support
6	“They were very helpful, even when I asked other staff who are not preceptors, they were supportive and answered my questions.”	
7	“Even when they are not the preceptors, they allow us to help and train us.”	
7	"They clarified and explained everything"	Clear explanations
2	"The CRN was amazing" “The CRN was very helpful with everything, she was the main source.”	Excellent CRN
3	“The CRN was the most encouraging thing, she asked us questions in the morning and then she came later in the afternoon to see if we discovered the answers and she discussed it with us.”	
9	“The CRN was the biggest support”	
5	“I was allowed to do some procedures like suctioning and IV cannula insertion and orogastric feeding.”	Practicing nursing skills
3	“I learn by action. The preceptor tells me the steps and I do them.”	
7	“I had the chance to insert NGT with my preceptor, it wasn’t correct at first, but the preceptor was very supportive and guided me step by step throughout the procedure. Then it felt very good and confident when I finally did it correctly.”	
8	“When they involve us in the procedures”	
2	“I learn by observing, I like to see the procedure first and then I start to do it.”	
8	“I learn by being hands-on”	
5	“I used to wake up every morning very excited because I wanted to know how my baby doing, if he is improving or not.”	Interesting cases
2	“Very critical and interesting cases like fractured skull, it made me want to look them up and read all about them and explained them to the physicians.”	
6	The number of common medications is not too much and too difficult to study, caffeine, Survanta, and other medications that are easy to understand	Graspable knowledge
4	“Babies’ mothers who thanked us and said nice words and prayers, this was very encouraging”	Family gratitude
7	“The rounds were also very encouraging. Whenever we attended rounds we found it very interesting and learned a lot about the cases”	Doctors rounds
2	“Especially when the consultants involve us in the rounds.”	
11	“One of the parts I really like during the day is the doctors round.”	
5	“When I’m asked to look for the answers, and I keep looking until I understand it. I learn best when I’m challenged. I would never forget it.”	Learning through challenge
1	“To have a specific assignment I would have to understand and study the whole case properly.”	
15	"One of the Arab preceptors used to teach me since the beginning of the shift the plan of the day and walks with me step by step. I was scared at the beginning and anxious but she took me by hand and encouraged me to hold the baby and do the cares."	Clear objectives and daily plans
14	"I liked it when the CRN looked at my objectives first and then she guided me to which patients would meet my objectives and help me fulfill my objectives. For example, she would say you can finish the blood transfusion in patient 1 and something else in patient 2. So this is the best way when they understand our objectives and help us finish them."	Objective-aligned patient care guidance

Theme 3: Barriers to learning in NICU

Navigating obstacles in NICU learning involves understanding the challenges nursing interns face, such as stress, limited hands-on experience, language barrier, accusations and blaming, negative environment, verbal abuse, racism, improper delegation, and unclear objectives. One major challenge is the inherent stress and emotional strain of the NICU environment. Long working hours coupled with exposure to critical situations and the fragility of the babies can be overwhelming for interns. Participant 4 mentioned, "Too much stress being interns," highlighting the demanding nature of the work. Another intern described the environment as depressing. Participant 5 said, "The environment is very depressing, and the manager keeps shouting."

Limited hands-on experience is another significant obstacle reported by interns. Some interns felt restricted in their ability to gain practical experience. Participant 1 shared, "I do not feel like I am practicing hands-on skills.” Communication barriers also pose challenges, especially when language or cultural differences exist between interns and staff. Participant 8 shared, "There was a barrier, the language and accents." Unclear objectives and task delegation can further impede an intern's progress. Feeling lost and unsure of what is expected can lead to anxiety and hinder the learning process. Participant 1 said, "We feel lost sometimes; we need to have clear objectives." 

Beyond the immediate workload and skill development challenges, some interns described an unsupportive work environment. This included experiences with accusations, verbal abuse, and even racism. Participant 1 recounted the stressful situation of being blamed after long shifts, "After working those 12 hours, we are accused of being cheaters!" Participant 14 described a culture of racial prejudice, "And they are very racist.” While interns acknowledged the importance of documentation, some felt that excessive paperwork detracted from valuable learning opportunities. Participant 8 stated, "But as an intern, I want to learn the fine motor skills of my future job, not to fill the papers." (Table 3).

**Table 3:** Barriers to learning faced by nursing interns in the NICU. NICU: neonatal intensive care unit.

Participant	Phrase	Code
8	"There was a barrier, the language and accents"	Language barrier
1	"It should be a closed area but it is a very open area" "Visitors and people keep coming inside"	Visitor interference
1	“After working those 12 hours we are accused of being cheaters!"	Accusations of cheating
8	“Because when we are accused of being cheaters they start to challenge us with different questions until they prove their points.”	
4	"Too much stress being interns”	Intern stress
5	“The environment is very depressing and the manager keeps shouting”	Negative environment
8	“It is not the sick babies, it is the place itself, with the dim lights and no colors.”	
8	“I don’t want to hear all those noises.”	
7	“I would like to take it as my elective rotation as well, but the environment is a bit toxic, there too many sick babies”	
7	“Some people cannot tolerate the sounds of crying babies and the devices and machines, it can be stressful.”	
6	“Verbal violence"	Verbal abuse
1	"I don’t feel like we are covering everything... we are only observing" "It’s a nice experience but I don’t feel like I’m practicing hands-on skills"	Lack of hands-on experience
1	“It affects us because they sometimes blame us for everything, and they don’t allow us to touch or do anything hands-on, so it affects our training, sometimes we are only sitting down doing nothing”	Accusations of being a source of infection
2	“They always point fingers at us for everything, they say that interns are the cause of infections”	
1	“We feel lost sometimes, we need to have clear objectives for each unit even if it’s verbal.”	Unclear training objectives
8	“We need them to keep track with our preceptors and teach them about our requirements.”	Insufficient faculty involvement
5	“we need to feel that someone is having our backs in the hospital.”	
7	“Using the interns as porters”	Improper delegations
2	“Improper delegations. For example, a nurse sent me to the PICU with an ABG sample and asked me to run the sample in their machine, without label. I was very scared that it would fall, or I would lose the sample anyway. When I reached the PICU, I was caught by infection control and they told me that I handled the sample in an unsafe way. It was traumatizing, it was my first time ever dealing with ABG.”	
4	“They use us for feeding all the babies sometimes. Sometimes we miss other opportunities to learn other things and we waste the time doing only feeding.”	
14	“One of the things that really really upsets me, is when they know that we are interns, they try to abuse us. For example, if they know that I’m capable of a certain procedure, they ask me to do it all the time, NGT insertion, they would say please sister insert the NGT I will go and eat. Or if I’m good at phlebotomy, then I become a lab technician, doing only phlebotomies all the time. They keep using us until the end of my rotation.” “Another thing that bothers me, that they treat us like porters, and if we don’t agree, they give us bad evaluation.”	
8	“But as an intern, I want to learn the fine and motor skills of my future job, not to fill papers”	Portfolio overload
3	“Portfolio t is a barrier rather than encouraging the learning process and training.”	
14	“And they are very racist.” “Too much racism on us Saudis.”	Racism
15	“They are not teaching us just because we are Saudis. When I enter the unit, the first thing I look for is an Arabic preceptor, because the rest would use me as porter or to bring her coffee.”	

Theme 4: Assessment and growth

Assessment and growth in NICU training focus on evaluating nursing interns' performance and development. This theme examines the evaluation methods, feedback mechanisms, and ongoing assessments that track interns' progress. Effective assessment practices are essential for fostering professional growth and ensuring high-quality care in the NICU.

The portfolio system, designed to document an intern's learning journey, appears to have limitations. Several interns expressed dissatisfaction with the portfolio, finding it unhelpful and time-consuming. Participant 8 stated, "The portfolio was no help at all.” Participant 10 said, "I do not want to be evaluated on the portfolio, I want to be evaluated on the cases that I handle, in a system just like all the nurses." Participant 1 suggested a simpler approach: "Make it only presentations and attendance." 

Timely and constructive feedback is another crucial element for growth. Interns benefit from regular evaluations that highlight their strengths and weaknesses, guiding improvement. Participant 1 highlighted the value of regular assessments: "Daily assessment on the same cases that we encounter during the shift. At the end of the day around 5:00 PM, come and ask me anything about the case." (Table 4).

**Table 4:** Assessment and growth of nursing interns in the NICU. NICU: neonatal intensive care unit.

Participant	Phrase	Code
8	“The portfolio was no help at all, we finished everything in it in the first week and then we started focusing on the training.”	Overwhelming paperwork
2	“We want to live the experience not to be filling papers.”	
3	“It is only extra pressure, and it is nothing like reality.”	
1	“Daily assessment on the same cases that we encounter during the shift. At the end of the day around 5:00 PM, come and ask me anything about the case.”	Suggestions
6	I suggest we only have an empty list and then we write the procedures that we finished.	
1	Make it only presentations and attendance, it is enough. The DOPs are very useless.	
11	I suggested before, we want a system for the students, same as the nurses.	
10	I don’t want to be evaluated on the portfolio, I want to be evaluated on the cases that I handle, in a system just like all the nurses.	
2	“They focus on the grading of the portfolio but never on our real performance.”	
14	“The evaluation is not fair. For example, she was evaluating me on drawing blood, and I prepared everything very well and did everything, when she evaluated me, she gave me a score of 1 in the consent criteria, how come? She is not even reading what she is evaluating me on.” “that they treat us like porters, and if we don’t agree, they give us bad evaluation.”	Unfair evaluation
3	“It is a waste of time and unfair grading.”	
9	“And sometimes when they ask me to do something alone, and then she gives me bad evaluation! She didn’t even see me doing it.”	

## Discussion

This study reveals four major themes regarding the nursing interns' experience in the NICU, including pleasant experiences, encouragement sources, learning barriers, and assessment and growth aspects.

Theme 1: Pleasant experiences in NICU training

Our study shows that nursing interns experience various pleasant moments during their NICU placements, including patient improvement, bonding with babies and families, appreciation, and unit-specific routines, e.g., quiet time and smooth endorsement. These positive experiences are consistent with the literature that emphasizes the rewarding aspects of neonatal care, such as witnessing the improvement and discharge of patients [12,15]. The emotional satisfaction derived from these moments contributes significantly to the interns' professional commitment and motivation. Positive clinical experiences are crucial in building nursing students' confidence and professional identity [21,22]. Nurses with a strong professional self-concept are more likely to demonstrate a positive attitude and high levels of work engagement, resulting in the delivery of high-quality patient care [23]. A study on the readiness of Saudi nursing interns (regardless of the unit where they demonstrate) underscores the importance of positive clinical experiences in fostering a sense of competence and professional commitment [24]. A recent study on the impact of quiet time on the psychological outcomes of NICU nurses in Saudi Arabia shows that many NICU nurses consider quiet time a power booster that helps them regain comfort [25]. Such experiences reinforce interns' motivation, suggesting that structured opportunities for interns to witness and participate in positive patient outcomes should be integral to training programs. Our study described the experience of nursing interns in the NICU, where pleasant moments come mainly from witnessing neonates’ improvement, discharge, interaction with families and babies, appreciation from preceptors, and some unit routines, e.g., quiet time and handover.

Theme 2: Sources of encouragement in NICU training

Support from NICU staff and families emerged as a crucial factor in encouraging and motivating nursing interns in their learning. Participants in this study received many forms of support from physicians, nurses, preceptors, CRN, nurse managers, and families. Research shows that a supportive clinical learning environment is crucial for achieving student learning outcomes, as it fosters the development of the skills, knowledge, and behaviors necessary to deliver safe, high-quality patient care [26-28]. Qualified nursing instructors can enhance students' learning by boosting their motivation, cultivating a positive attitude toward the profession, sparking greater interest in patient care, encouraging active participation in care activities, and promoting academic success. This can be achieved through offering constructive feedback and fostering a supportive learning environment [29,30]. Our study adds that the CRN plays a significant role in nursing interns’ learning in the NICU. The role of the CRN in providing constructive feedback and aligning patient care tasks with learning objectives was particularly appreciated by interns, reflecting the value of structured mentorship in clinical training. Encouragement from experienced nurses and mentors helps interns feel valued and capable, thereby enhancing their learning experiences and professional growth. A study conducted in Saudi Arabia assessed the importance of preceptorship programs for enhancing nursing interns learning in clinical settings. The study found the preceptorship program significantly enhanced nursing interns' ability to manage real patient care in clinical settings. As such, it serves as an effective teaching strategy that helps novice nurses progress to the next level of clinical competence. Most nursing interns who participated in the study regarded the preceptorship as a valuable experience. They believed that the preceptor's availability, approachable demeanor, and reliability were key factors in enhancing their clinical competence [17].

Theme 3: Barriers to effective learning in NICU

In our study, nursing interns faced significant barriers during their NICU training despite the positive aspects. Improper delegation of tasks, less hands-on practice, discouraging environment, overload of portfolio and paper requirements, and mistrust in nursing interns were major challenges that hindered effective learning. Clinical practice is crucial for acquiring nursing skills, yet nursing interns in the NICU often have limited opportunities for hands-on practice. This finding is consistent with a study conducted in South Korea that found the main challenges hindering students' learning in the NICU are the lack of practical opportunities, inadequate instruction, and the gap between theory and practice [14]. A qualitative study from Saudi Arabia explored the factors that contributed to successful clinical internships in private and public hospitals, highlighting some barriers for internship students regardless of the unit where they practiced. The main barriers included exploitation of the nursing interns, low self-confidence, lack of motivation, and the lengthy duration of the programs [6]. In our study, the main barriers for internship students to learn in the NICU were an improper delegation of tasks, mistrust in nursing interns, and an overload of portfolio requirements, highlighting the need for the creation of a more effective clinical internship program that considers the aforementioned barriers.

Theme 4: Assessment and growth (evaluative insights in NICU training)

Our findings prominently emphasized the importance of regular assessment and feedback in facilitating interns' learning growth. Daily evaluations and constructive feedback from supervisors help nursing interns identify their strengths and areas for improvement, fostering continuous professional development. This is consistent with the findings of a study conducted in Saudi Arabia that emphasized the need for ongoing assessment to build nursing interns' self-efficacy and readiness for professional practice [24]. Assessment practices and daily feedback are crucial for intern development. Regular and constructive feedback helps interns identify their strengths and areas for improvement, fostering continuous professional development [24].

The findings of this study hold significant implications for improving nursing internship programs, particularly within the context of the NICU in Saudi Arabia. It is imperative to establish structured internship programs with clear objectives to guide interns throughout their training and for interns to be aware of these objectives and how they can meet them. Additionally, there is a pressing need for proper training of preceptors who play a crucial role in mentoring and supervising interns. Fostering a culture of positive encouragement within internship programs is paramount. Regularly praising interns for their achievements and efforts reinforces a sense of accomplishment and professional growth, along with providing feedback. Furthermore, maximizing opportunities for hands-on experience, whenever possible and safe for the babies, enhances interns' confidence and competence in clinical practice. Additionally, ensuring clear explanations from preceptors to interns during training, including detailed step-by-step instructions when performing procedures, facilitates effective learning and skill acquisition. The overwhelming paperwork identified as a barrier to effective training highlights the need for streamlining administrative processes within internship programs.

Despite the valuable insights gained from this study, several limitations should be acknowledged. Firstly, the qualitative nature of the research introduces subjectivity in interpreting the results. While efforts were made to ensure rigor and reliability in the analysis process, the inherent subjectivity of qualitative research may influence the findings. Secondly, the data for this study were collected from a single setting, from one private hospital in Jeddah, Saudi Arabia. Consequently, the generalizability of the findings to other contexts or populations may be limited. However, other hospitals and nursing schools in Saudi Arabia can consider the points raised by this study when they place nursing interns in the NICU. Future research should explore nursing interns' experiences across various healthcare settings to understand their training needs better.

## Conclusions

This study provides valuable insights into the experiences of nursing interns in the NICU in Saudi Arabia. Positive experiences, such as patient recovery and encouragement from preceptors, play a crucial role in enhancing nursing interns' motivation and professional growth in the NICU. However, significant barriers, including improper task delegation and limited hands-on practice, hinder effective learning. Regular assessment and feedback further contribute to the interns’ development and readiness for professional practice. Nursing internship programs in the NICU require a collective effort from nursing educators, managers, preceptors, and staff in the NICU to enhance nursing interns' learning experiences, considering the positive and negative contributing factors identified in this study.
